# Prevalence of Health Care Worker Burnout During the Coronavirus Disease 2019 (COVID-19) Pandemic in Japan

**DOI:** 10.1001/jamanetworkopen.2020.17271

**Published:** 2020-08-04

**Authors:** Takahiro Matsuo, Daiki Kobayashi, Fumika Taki, Fumie Sakamoto, Yuki Uehara, Nobuyoshi Mori, Tsuguya Fukui

**Affiliations:** 1 Department of Infectious Diseases, St Luke’s International Hospital, Tokyo, Japan; 2 Department of General Internal Medicine, St Luke’s International Hospital, Tokyo, Japan; 3 Graduate School of Public Health, St Luke’s International University, Tokyo, Japan; 4 Department of Nephrology, St Luke’s International Hospital, Tokyo, Japan; 5 Quality Improvement Center, St Luke’s International Hospital, Tokyo, Japan; 6 Department of Clinical Laboratory Center, St Luke’s International Hospital, Tokyo, Japan

## Abstract

This cross-sectional study evaluates the prevalence of and factors associated with burnout among frontline health care workers during the coronavirus disease 2019 (COVID-19) pandemic in Japan.

## Introduction

The coronavirus disease 2019 (COVID-19) pandemic has placed considerable psychological strain on frontline health care workers (HCWs).^1^ Although the problem of burnout, which overlaps with the symptoms of depression,^2^ remains urgent, few studies have addressed it comprehensively. The objective of this study was to evaluate the prevalence of burnout among frontline HCWs during the COVID-19 pandemic in Japan based on job categories and other factors.

## Methods

We conducted an online cross-sectional survey among HCWs between April 6 and April 19, 2020, at St Luke’s International Hospital, a tertiary hospital in Tokyo, Japan, with among the highest numbers of patients with COVID-19 in the country. We selected HCWs, including physicians, nurses, laboratory medical technologists, radiological technologists, and pharmacists, who worked in departments in which they had contact with patients with COVID-19, including emergency departments, general internal medicine departments, respiratory medicine departments, infectious disease departments, general wards, and intensive care units. An explanation of the sample size calculation appears in the eAppendix in the Supplement. This study was approved by the institutional review board of St Luke’s International Hospital in Tokyo, Japan. A letter of informed consent was distributed to the participants via email, and completion of the questionnaire implied their consent. This study followed the Strengthening the Reporting of Observational Studies in Epidemiology (STROBE) reporting guideline.

The web-based survey was generated using SurveyMonkey, a cloud-based survey development application. The survey solicited responses regarding participants’ demographic characteristics (age and gender), professional history (job category and years of experience), working environment characteristics (mean weekly working hours, days off per month, and hours of sleep per day), types of anxiety perceived, changes compared with before the pandemic, and types of support needed.

The primary outcome was the prevalence of burnout among frontline HCWs in departments with direct contact with patients with COVID-19, using the validated Japanese version of the Maslach Burnout Inventory–General Survey,^3^ which is currently considered the criterion standard for measuring burnout. This 16-item questionnaire contains 3 subscales that evaluate what are considered the 3 major domains of burnout, ie, emotional exhaustion, cynicism (ie, depersonalization), and professional efficacy (ie, personal accomplishment). High levels of exhaustion (>3.5) plus either high cynicism (>3.5) or low professional efficacy (<2.5) were selected as the primary criteria for burnout.

We first compared the baseline characteristics of those who did and did not have burnout by using the χ^2^ difference test for categorical variables and the Mann-Whitney U test for continuous variables. Given the limited information available on confounding by potential risk factors for burnout, we used logistic regression analyses to evaluate whether any factors, such as types of anxiety perceived, changes compared with the prepandemic period, and types of support needed, were significant by integrating data on the participants’ backgrounds into the model. All analyses were performed using SPSS statistical software version 19.0 (IBM Corp) with 2-tailed significance set at *P* < .05.

## Results

Of 488 HCWs, 369 (75.6%) responded to the survey, of whom 57 (15.4%) were excluded because of missing data. The final sample included 312 respondents, with a median (interquartile range [IQR]) age of 30.5 (26-40) years, 223 (71.5%) women, and median (IQR) experience of 7.0 (3-15) years. The overall burnout prevalence was 31.4% (98 of 312). Of 126 nurses, 59 (46.8%) were experiencing burnout; of 22 radiological technologists, 8 (36.4%) were experiencing burnout; and of 19 pharmacists, 7 (36.8%) were experiencing burnout (Table 1). Table 1 shows that the burnout group had a significantly higher percentage of women (79 [80.6%] vs 144 [67.%]; *P* = .02), fewer median (IQR) days off per month (8 [6-9.3] days vs 9 [8-10] days; *P* = .03), and more respondents with intentions of dropping out (73 [74.5%] vs 52 [24.3%]; *P* = .01), along with significantly lower median (IQR) age (28 [25-34] years vs 32 [27-43] years; *P* = .001) and years of experience (5 [2-8] years vs 8 [3-18] years; *P* = .001) compared with the group without burnout. After adjusting for potential covariates and using physicians as the comparison group, burnout prevalence was significantly higher among nurses (OR, 4.9; 95% CI, 2.2-11.2; *P* = .001), laboratory medical technologists (OR, 6.1; 95% CI, 2.0-18.5; *P* = .002), radiological technologists (OR, 16.4; 95% CI, 4.3-61.6; *P* = .001), and pharmacists (OR, 4.9; 95% CI, 1.3-19.2; *P* = .02). Also, burnout was more prevalent in participants with fewer years of experience (OR, 0.93; 95% CI, 0.89-0.97; *P* = .001), with heightened anxiety because of unfamiliarity with personal protective equipment (OR, 2.8; 95% CI, 1.4-5.5; *P* = .002), with decreased sleep length compared with the prepandemic period (OR, 2.0; 95% CI, 1.1-3.6; *P* = .03), with the desire for reduced workloads (OR, 3.6; 95% CI, 1.6-8.0; *P* = .002), and with the desire for expectations of appreciation or respect (OR, 2.2; 95% CI, 1.1-4.6; *P* = .03) (Table 2).

**Table 1.** Comparison of Demographic Characteristics of Participants With and Without Burnout

Characteristic	No. (%)			*P* value
	With burnout (n = 98)	Without burnout (n = 214)	Overall (N = 312)	
Women	79 (80.6)	144 (67.3)	223 (71.5)	.02
Age, median (IQR), y	28 (25-34)	32 (27-43)	30.5 (26-40)	.001
Occupation				
Physician	11 (11.2)	71 (33.2)	82 (26.3)	.001
Nurse	59 (60.2)	67 (31.3)	126 (40.4)	
Laboratory medical technologist	13 (13.3)	50 (23.4)	63 (20.2)	
Radiological technologist	8 (8.2)	14 (6.5)	22 (7.1)	
Pharmacist	7 (7.1)	12 (5.6)	19 (6.1)	
Experience, median (IQR), y	5 (2-8)	8 (3-18)	7 (3-15)	.001
Amount of contact with patients with COVID-19, median (IQR), d/wk	3 (1-5)	3 (1-5)	3 (1-5)	.70
Work per week, median (IQR), h	63 (56-70)	59.5 (50-70)	61 (55.3-70)	.12
Sleep duration per night, median (IQR), h	6 (5-7)	6 (5-7)	6 (5-7)	.87
Time off per month, median (IQR), d	8 (6-9.3)	9 (8-10)	8 (7-10)	.03
Affected by social media, median (IQR), No.	54 (39-77)	53.4 (39-70)	54 (39-70)	.27
Dropout intentions	73 (74.5)	52 (24.3)	125 (40.1)	.01
Type of anxiety perceived				
Getting COVID-19	92 (93.9)	185 (86.9)	277 (89.1)	.08
Transmission to family members	85 (87.6)	167 (78)	252 (80.8)	.06
Transmission to coworkers and friends	90 (91.8)	178 (83.6)	268 (86.2)	.05
Transmission to patients	83 (84.7)	159 (74.3)	242 (77.6)	.04
Unfamiliarity with PPE	87 (88.8)	163 (76.2)	250 (80.1)	.009
Lack of daily necessities	81 (82.7)	143 (66.8)	224 (71.8)	.004
Childcare	15 (15.3)	44 (20.7)	59 (19.0)	.28
Changes compared with prepandemic period				
Increased workload	65 (68.4)	96 (47.3)	161 (54.0)	.001
Unhealthy diet	50 (52.6)	75 (36.9)	125 (41.9)	.01
Decreased sleep length	47 (49.5)	56 (27.6)	103 (34.6)	.001
Increased drinking of alcohol	23 (24.2)	35 (17.2)	58 (19.5)	.16
Decreased relaxation time	70 (73.7)	114 (56.2)	184 (61.7)	.005
Types of support needed now				
Workload reduction	80 (84.2)	102 (51)	182 (61.7)	.001
Staff increase	70 (73.7)	116 (58)	186 (63.1)	.01
Expectation of appreciation or respect	77 (81.1)	105 (52.5)	182 (61.7)	.001
Hazard pay	85 (89.5)	150 (75)	235 (79.7)	.003
Educational resources for infection prevention	61 (64.2)	96 (48)	157 (53.2)	.01
Childcare support	57 (60)	109 (54.5)	166 (56.3)	.38
Counseling	47 (49.5)	61 (30.5)	108 (36.6)	.002

Abbreviations: COVID-19, coronavirus disease 2019; IQR, interquartile range; PPE, personal protective equipment.

**Table 2.** Factors Associated With Burnout

Factor	OR (95% CI)	*P* value
Occupation		
Physician	1 [Reference]	NA
Nurse	4.9 (2.2-11.2)	.001
Laboratory medical technologist	6.1 (2.0-18.5)	.002
Radiological technologist	16.4 (4.3-61.6)	.001
Pharmacist	4.9 (1.3-19.2)	.02
Years of experience	0.93 (0.89-0.97)	.001
Anxiety because of unfamiliarity with PPE	2.8 (1.4-5.5)	.002
Decreased sleep	2.0 (1.1-3.6)	.03
Desire for reduced workload	3.6 (1.6-8.0)	.002
Desire for expectations of appreciation or respect	2.2 (1.1-4.6)	.03

Abbreviations: NA, not applicable; OR, odds ratio; PPE, personal protective equipment.

## Discussion

In this study, we found that more than 40% of nurses and more than 30% of radiological technologists and pharmacists met the criteria for burnout. To our knowledge, this was the first report on burnout comparing job categories and associated risk factors among HCWs in Japan during a pandemic. The explanation for the higher prevalence of burnout among nonphysicians could be that these job categories have lower dimensions of control (skill discretion and decision authority)^4^ compared with physicians. Also, the desire for expectations of appreciation or respect, 1 of the social supports (ie, from supervisor, coworker, and others), may be an important variable in studies exploring the association between job characteristics and burnout.^4^ It is essential that team leaders and peers appreciate members’ dedicated work through positive messages of gratitude and support.^5^

This study has limitations. It was conducted in a single institution focusing on only frontline departments providing care or services to patients with COVID-19. The findings of this study may not be generalizable to other countries or regions. Furthermore, because we have not assessed the baseline level of burnout before the pandemic, we were unable to compare changes in prevalence. Further studies focusing on both identification and interventions for frontline HCWs to prevent and reduce risk of burnout are needed.

## Notes

Supplement. **eAppendix. **Sample Size Calculation
