# Supplementary material for: Prevalence of Health Care Worker Burnout During the Coronavirus Disease 2019 (COVID-19) Pandemic in Japan
Source: JAMA Netw Open. 2020 Aug 4;3(8):e2017271. doi: 10.1001/jamanetworkopen.2020.17271 (PMC7403916; doi:10.1001/jamanetworkopen.2020.17271)
Supplement: Supplement. — eAppendix. Sample Size Calculation [file jamanetwopen-3-e2017271-s001.pdf]

# Supplementary Online Content

Matsuo T, Kobayashi D, Taki F, et al. Prevalence of health care worker burnout during the coronavirus disease 2019 (COVID-19) pandemic in Japan. *JAMA Netw Open*. 2020;3(8):e2017271. doi:10.1001/jamanetworkopen.2020.17271

**eAppendix.** Sample Size Calculation

This supplementary material has been provided by the authors to give readers additional information about their work.

© 2020 Matsuo T et al. *JAMA Network Open*.

**eAppendix.** Sample Size Calculation

When we calculate sample size to compare the rates of burnout between physicians and other healthcare workers, we assumed the rate among physicians and that among healthcare workers as 15% and 25% respectively. Defining $\alpha$ and $\beta$ as 0.05 and 0.20, respectively, at least 121 participants were required for one arm. In addition, considering to perform logistic regression with 6 variables in the model, approximately 60 burnout were required. Based on these two approached, we considered at least 300 participants were required. Therefore, in terms of comparison of the rates in each healthcare workers other than physician, sample size might be insufficient.

© 2020 Matsuo T et al. *JAMA Network Open*.
